# Modified Hammerstein-Like Hysteresis Modeling and Composite Control Methods for Fast Steering Mirrors

**DOI:** 10.3390/mi16060626

**Published:** 2025-05-26

**Authors:** Kairui Cao, Zekun Li, Guanglu Hao, Rui Li, Jie Zhang, Jing Ma

**Affiliations:** 1 National Key Laboratory of Tunable Laser Technology, Harbin Institute of Technology, Harbin 150001, China; cao-kairui@hit.edu.cn (K.C.); 21b921012@stu.hit.edu.cn (G.H.); 22b921011@stu.hit.edu.cn (J.Z.); majing@hit.edu.cn (J.M.); 2 College of Control Science and Engineering, Zhejiang University, Hangzhou 310027, China; rlihit@hotmail.com

**Keywords:** fast steering mirrors, hysteresis nonlinearity, rate-dependent model, Hammerstein model, composite control

## Abstract

Fast steering mirrors (FSMs), actuated by piezoelectric ceramics, play pivotal roles in satellite laser communication, distinguished by their high bandwidth and fast responsiveness, thereby facilitating the precise pointing and robust tracking of laser beams. However, the dynamic performance of FSMs is notably impaired by the hysteresis nonlinearity inherent in piezoelectric ceramics. Under dynamic conditions, rate-dependent hysteresis models and Hammerstein models are predominantly employed to characterize hysteresis nonlinearity. By combining the advantages of these two models, a hysteresis model termed modified Hammerstein-like (MHL) model is proposed. This model integrates an input time delay, a rate-dependent hysteresis term, and a linear dynamic term in a cascaded structure, effectively capturing the dynamic characteristics of hysteresis systems across a broad frequency range. Additionally, a composite control strategy is tailored for the MHL model which consists of a feedforward compensator based on a rate-dependent hysteresis inverse model and a proportional–integral (PI) controller for closed-loop regulation. Experimental results demonstrate the effectiveness of the proposed modeling and composite control methods.

## 1. Introduction

**Figure 1:**
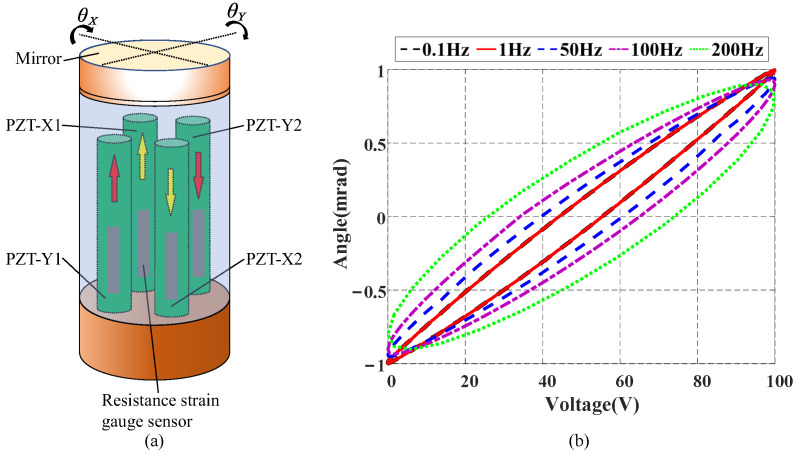
(**a**) Schematic diagram of FSM. (**b**) Diagram of rate-dependent hysteresis.

Piezoelectric ceramic actuators, characterized by their rapid response and high stiffness, have been widely used in many fields, such as satellite laser communication [1]. In the beam control system of satellite laser communication, fast steering mirrors (FSMs), driven by piezoelectric ceramics, enable the high-precision pointing and tracking of the laser beam [2]. In an FSM, four piezoelectric ceramics are arranged in a cross shape. It has two tilt axes, with two ceramics opposing each other forming a pair to control the deflection of the FSM along one direction, as shown in Figure 1a. However, the hysteresis nonlinearity of piezoelectric ceramics significantly degrades the dynamic performance of FSMs. The hysteresis characteristics in FSMs are primarily manifested as a nonlinear relationship between the control voltage and deflection angle. Furthermore, the hysteresis nonlinearity varies with the increase in input frequency, as shown in Figure 1b. To mitigate the hysteresis effect, it is important to establish accurate hysteresis models and design corresponding compensation controllers.

There are primarily two types of hysteresis models for low-frequency input signals: (1) physical models and (2) phenomenological models. Physical models seek to characterize hysteresis nonlinearity by incorporating the fundamental physical mechanisms of piezoelectric materials [3,4]. However, due to the inherent complexity of these mechanisms and the challenges in generalizing them to different materials, physical models have been relatively less studied and reported in the literature. In contrast, phenomenological models employ appropriate mathematical models to describe hysteresis nonlinearity, resulting in behavior similar to physical models, but without explicitly accounting for the physical mechanisms of piezoelectric materials [5,6,7,8]. Phenomenological models such as the Bouc–Wen model [9,10], the Duhem model [11,12], the Preisach model [13], the Prandtl–Ishlinskii model [14,15], the Maxwell model [16], and other models [17,18] have been developed to describe hysteresis nonlinearity. The Bouc–Wen model and Duhem model belong to differential equation-based models, which use differential equations to model the hysteresis nonlinearity. The Preisach model, Prandtl–Ishlinskii model, and Maxwell model fall into the category of operator-based models, where the weighted superposition of simple hysteresis operators is used to describe hysteresis nonlinearity. A phenomenological hysteresis model based on Madelung’s rules is proposed in [19,20]. Different from the differential equation-based and operator-based models, this method focuses on describing the hysteresis curves directly. It is noted that all the aforementioned models are presented for low-frequency inputs. These models are referred to as static hysteresis models or rate-independent hysteresis models.

As shown in Figure 1b, the hysteresis loop gradually widens with the increase in input frequency, making rate-independent hysteresis models unable to describe the variations accurately. Some scholars believe that the change in the shape of the hysteresis loop is caused by the increase in input frequency. Thus, the derivative of the input $v\left(t\right)$ is introduced to rectify the shape change [7,21,22,23]. This type of model, which introduces the input rate correction term into the rate-independent model, is commonly referred to as the rate-dependent hysteresis model. Other scholars consider that the phenomenon of the hysteresis loop shape varying with frequency arises from more complex causes. To account for this, a rate-independent hysteresis in series with a linear dynamic term is employed to describe such variations in the hysteresis loop. This model is also known as the Hammerstein model [24,25] and has been widely applied in hysteresis modeling. In [26,27,28,29], the Hammerstein-like model is proposed, where the effect of the input time delay is taken into account.

Generally, it is difficult to compare rate-dependent models and Hammerstein models. However, to illustrate the main idea of this paper, we attempt to summarize the characteristics of both models. A rate-dependent model often possesses an inverse model, which can be directly utilized for feedforward compensation and achieve input–output linearization within a certain frequency range. In contrast, due to the presence of the linear dynamic term in the Hammerstein model, it lacks an inverse model and cannot realize full feedforward compensation (only the rate-independent term can be compensated). However, the Hammerstein model outperforms the rate-dependent model in describing the frequency characteristics of the system, particularly in the high-frequency range. Combining the advantages of both models could potentially yield better results in modeling, feedforward compensation, and closed-loop control, which is precisely the main idea of this paper. The primary contributions of our methods are two-fold:

1. A modified Hammerstein-like (MHL) hysteresis modeling approach is proposed; it integrates an input time delay, a rate-dependent hysteresis term, and a linear dynamic term in a cascaded structure. As we will see in the experiments, the presented model can effectively capture the dynamic characteristics of an FSM across a wide frequency range;
2. A composite control method tailored for the MHL model is introduced. The presented controller consists of two parts: a feedforward compensator and a traditional proportional–integral (PI) controller. The feedforward compensator is the inverse of the rate-dependent term in the MHL model, while the PI controller is used for closed-loop regulation.

The subsequent chapters are organized as follows: The MHL model and its identification method are introduced in Section 2. Section 3 showcases the composite control method of the MHL model. In Section 4, experiments are conducted to show the effectiveness of the presented methods. The conclusion is drawn in Section 5.

## 2. Modified Hammerstein-Like (MHL) Model and Its Identification Method

### 2.1. Rate-Independent Hysteresis Model Based on Madelung’s Rules

**Figure 2:**
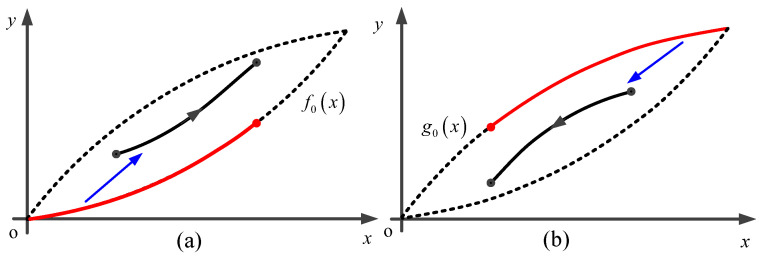
Schematic diagram of description method for hysteresis curve. (**a**) Ascending curve; (**b**) Descending curve.

In the early 20th century, German physicist Madelung summarized hysteresis phenomena and proposed several rules that later bore his name. Madelung’s rules are inherently satisfied in all the aforementioned differential equation-based and operator-based hysteresis models. In our previous studies [19,20], a rate-independent model was developed based on Madelung’s rules. This model comprises two parts: The first part is an algorithm that converts Madelung’s rules, described in natural language, into a computer program to automatically implement the wiping-out mechanism [30,31]. The proposed model naturally satisfies Madelung’s rules. The second part concerns the description method for hysteresis curves. Based on the similarities between internal hysteresis loops and the major hysteresis loops, we utilize the ascending and descending curves of the major hysteresis loop to describe the hysteresis curves, as shown in Figure 2. The major ascending curve $f_{0}\left(x\right)$ and descending curve $g_{0}\left(x\right)$ can be mathematically expressed as

(1) $$f_{0}\left(x\right)=p_{5}x^{5}+p_{4}x^{4}+p_{3}x^{3}+p_{2}x^{2}+p_{1}x$$

(2) $$g_{0}\left(x\right)=q_{5}x^{5}+q_{4}x^{4}+q_{3}x^{3}+q_{2}x^{2}+q_{1}x$$

It is worth pointing out that the rate-independent model only requires identifying the parameters of the major hysteresis curves $f_{0}\left(x\right)$ and $g_{0}\left(x\right)$. The forms of Equations (1) and (2) are not limited to polynomials only, and they can also be represented by other forms of functions, such as piecewise linear functions.

### 2.2. Rate-Dependent Hysteresis Model

**Figure 3:**
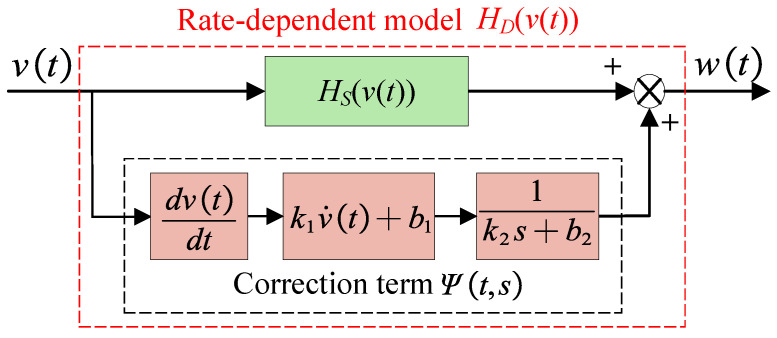
Schematic diagram of rate-dependent hysteresis model.

On the basis of the Madelung model, a rate-dependent term $\Psi \left(t,s\right)$ is introduced to correct the effect caused by the increase in input frequency [23], as shown in Figure 3. The rate-dependent model is composed of the Madelung model and a rate-dependent correction term in parallel. Mathematically, the hysteresis model $H_{D}\left(v\left(t\right)\right)$ can be described as follows:

(3) $$H_{D}\left(v\left(t\right)\right)=H_{S}\left(v\left(t\right)\right)+\Psi \left(t,s\right)$$

(4) $$\Psi \left(t,s\right)=\left(k_{1}\dot{v}\left(t\right)+b_{1}\right)⊙\frac{1}{k_{2}s+b_{2}}$$

where $H_{S}\left(v\left(t\right)\right)$ is the rate-independent model based on Madelung’s rules, and its major hysteresis curves can be represented by Equations (1) and (2). $\Psi \left(t,s\right)$ is the rate-dependent correction term, which is composed of two modules connected in series (⊙ is the cascading symbol). $\dot{v}\left(t\right)$ is the derivative of the input, *s* is the Laplace operator, and $k_{1}$, $b_{1}$, $k_{2}$, and $b_{2}$ are parameters to be identified.

### 2.3. MHL Model

According to basic control theory, it is evident that the final value of the correction term $\Psi \left(t,s\right)$ of $H_{D}\left(v\left(t\right)\right)$ approaches $k_{1}/k_{2}$ as the input frequency tends to infinity, which is not consistent with the actual situation (as the output of the system should be 0 under infinite input frequency excitation). Therefore, the rate-dependent model in [23] is not adequate for describing the high-frequency characteristics of the hysteresis system.

**Figure 4:**
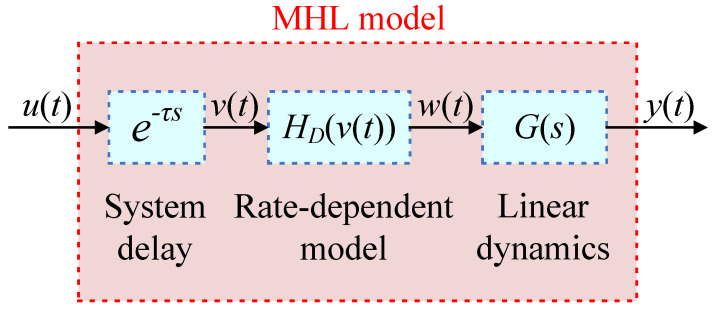
MHL model structure schematic diagram of FSM system.

Since the Hammerstein model includes a dynamic term $G\left(s\right)$, it can accurately describe the dynamic characteristics of the system in the high-frequency region. Inspired by the Hammerstein model, we can attempt to add $G\left(s\right)$ after the rate-dependent model $H_{D}\left(v\left(t\right)\right)$ to enhance its modeling accuracy in the high-frequency range. Furthermore, by introducing the time delay term from the Hammerstein-like model [26,27,28,29], a novel hysteresis model is proposed as shown in Figure 4, where $v\left(t\right)$ and $w\left(t\right)$ are unmeasurable. The introduction of a time delay can reduce the order of the linear system and alleviate the issue of inaccurate high-frequency descriptions. This model consists of an input time delay, a rate-dependent term, and a dynamic term cascaded in series, and it is named the modified Hammerstein-like (MHL) model. The output of the model can be expressed as

(5) $$y\left(t,s,\tau \right)=u\left(t\right)\cdot \left(e^{-\tau s}⊙H_{D}\left(v\left(t\right)\right)⊙G\left(s\right)\right)$$

In [26,27,28,29], the time delay term appears at the end of the model and is considered to be generated by the sensor. The FSM employs a resistance strain gauge sensor, which has a very high bandwidth and is not the main cause of the time delay. We believe that this time delay is caused by the relaxation effect inside the piezoelectric ceramic and therefore place the time delay term at the front of the MHL model.

### 2.4. Parameter Identification

**Figure 5:**
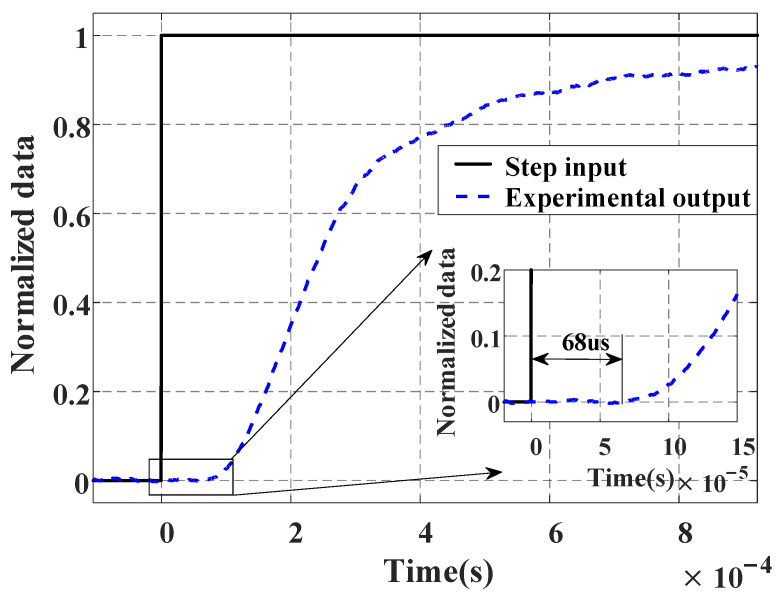
Step response of time delay phenomenon.

Since the MHL model is a cascade structure of three parts, the parameters can be identified sequentially. (1) The time delay $e^{-\tau s}$ is obtained by measuring the step response of the FSM system, as shown in Figure 5. (2) By employing the identification method presented in [23], the rate-dependent model $H_{D}\left(v\left(t\right)\right)$ can be established. (3) Based on the least square method, we take the output $w\left(t\right)$ of the rate-dependent term as the input and the actual output $y\left(t\right)$ as the output to identify $G\left(s\right)$. It is worth noting that $w\left(t\right)$ is a virtual variable which is the output of the actual input signal $u\left(t\right)$ after passing through the time delay and rate-dependent term. Therefore, it is necessary to first identify τ and $H_{D}\left(v\left(t\right)\right)$.

## 3. Composite Control Method

**Figure 6:**
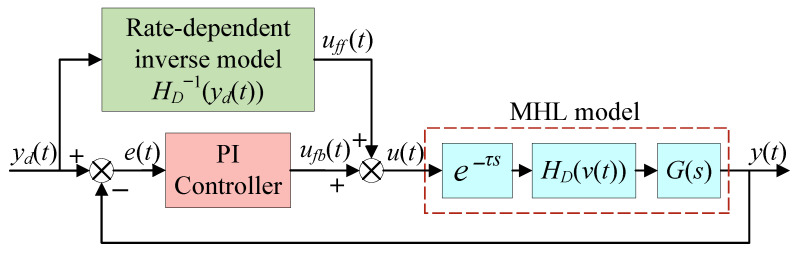
Schematic diagram of proposed composite control method.

In this section, a composite control method is tailored for the established MHL model, as illustrated in Figure 6. This composite controller consists of two components: a feedforward compensator and a traditional proportional–integral (PI) controller. The feedforward compensator is the inverse of the rate-dependent term $H_{D}\left(v\left(t\right)\right)$, with a structure similar to that shown in Figure 3, and its output is

(6) $$u_{ff}\left(t\right)=H_{S}^{-1}\left(y_{d}\left(t\right)\right)+\Psi^{\prime }\left(t,s\right)$$

(7) $$\Psi^{\prime }\left(t,s\right)=\left(k_{1}^{\prime }{\dot{y}}_{d}\left(t\right)+b_{1}^{\prime }\right)⊙\frac{1}{k_{2}^{\prime }s+b_{2}^{\prime }}$$

where $H_{S}^{-1}\left(y_{d}\left(t\right)\right)$ is the rate-independent inverse model based on Madelung’s rules. $\Psi^{\prime }\left(t,s\right)$ is the rate-dependent correction term with a structure similar to Equation (4). $k_{1}^{\prime }$, $b_{1}^{\prime }$, $k_{2}^{\prime }$, and $b_{2}^{\prime }$ are parameters to be identified.

The PI controller is employed in the feedback loop, which can be expressed as

(8) $$u_{fb}\left(t\right)=k_{p}+k_{i}\int_{0}^{t}e\left(\sigma \right)d\sigma$$

where $k_{p}$ and $k_{i}$ are the proportional and integral coefficients, respectively, and $e\left(t\right)$ is the tracking error.

**Figure 7:**
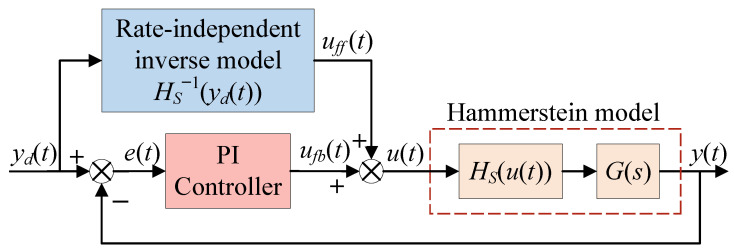
Schematic diagram of traditional composite control method.

Since the Hammerstein model incorporates a dynamic term $G\left(s\right)$, it is generally used to describe the high-frequency dynamic behavior of the hysteresis system. However, due to the lack of an inverse model for this linear dynamic term, the Hammerstein model cannot directly implement feedforward compensation. In order to eliminate hysteresis nonlinearity and achieve better control performance, the traditional composite control method, as shown in Figure 7, is commonly applied to the hysteresis system described by the Hammerstein model. This controller typically employs a rate-independent hysteresis inverse model as the feedforward compensator and uses a PI controller with the same structure as Equation (8) for closed-loop regulation. The expression for the output of the feedforward compensator is as follows:

(9) $$u_{ff}\left(t\right)=H_{S}^{-1}\left(y_{d}\left(t\right)\right)$$

This compensator can only diminish rate-independent hysteresis nonlinearity, and it is difficult to further expand the compensation range except for achieving input–output linearization in the low-frequency range. The dynamic characteristics of the entire system still need to be handled by the PI controller, which undoubtedly increases its workload. Additionally, for input signals with higher frequencies, the traditional composite control method will not be able to achieve the desired tracking accuracy.

Unlike the Hammerstein model, we introduce a rate-dependent model $H_{D}\left(v\left(t\right)\right)$ in the MHL modeling process, which often possesses an inverse model that can be directly utilized for feedforward compensation. By replacing the rate-independent inverse model (see Equation (9)) in traditional composite control methods with a rate-dependent inverse model (see Equation (6)), a novel composite control method is established. Such a modification aids in compensating for more system dynamics that can be accurately modeled, thereby expanding the compensation range and enhancing the control performance of the closed-loop system. After compensation using the rate-dependent inverse model, the hysteresis system achieves input–output linearization within a certain frequency range (within 100 Hz), reducing the workload of the feedback controller. The introduction of the rate-dependent term allows for a reduction of up to 40% in the output of the PI feedback controller while maintaining the same closed-loop tracking accuracy. Conversely, with the same PI feedback controller output, the closed-loop tracking error is decreased by up to 80%. Reducing the feedback controller’s workload effectively decreases the control output amplitude, avoiding excessive responses or high-frequency oscillations. At the same time, it reduces adjustment cycles, not only shortening the system’s response time but also enhancing the closed-loop bandwidth. Additionally, it notably eliminates hysteresis effects, helping to minimize the steady-state error of the closed-loop system. These improvements collectively contribute to a substantial enhancement in the overall performance of the composite control method.

## 4. Experimental Verification

### 4.1. Experimental Setup

**Figure 8:**
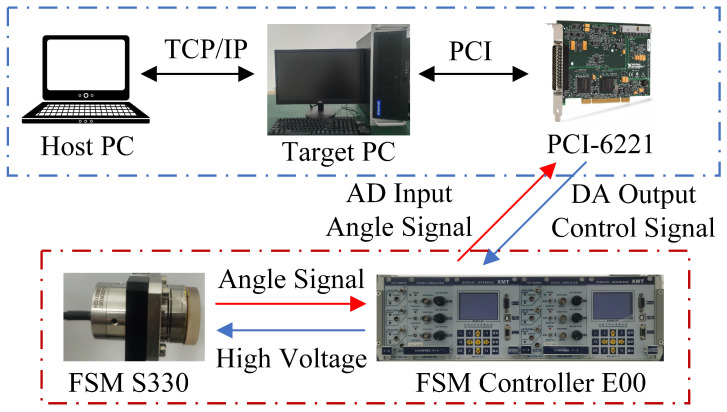
Experimental setup.

The platform depicted in Figure 8 comprises a host computer, a target computer, an FSM, a PCI-6221 data acquisition and control card, and its E00 high-voltage driver. The experiment employs the FSM (S330.2SL) manufactured by PI Corporation for parameter identification, model validation, and tracking performance testing. The LabVIEW real-time operating system manages real-time control and the sampling process, with the PCI-6221 handling the 16-bit analog-to-digital conversion. The DA output control signal of PCI-6221 is amplified by the E00 high-voltage driver to drive the FSM, with a fixed gain of 20 for E00. Additionally, a resistance strain gauge sensor (SGS) is connected to the PZT actuators of the FSM for angle measurement; the SGS signals are then sampled through the first AD channel of PCI-6221 post-amplification by E00.

### 4.2. Parameter Identification Results

In subsequent experiments, the normalized root mean square error (NRMSE) and normalized maximum error (NME) are selected as unified performance evaluation indicators. Among them, $y_{ai}$ and $y_{i}$ represent the actual angle and predicted angle of the FSM system, respectively, and *M* denotes the number of samples.

(10) $$NME=\frac{\max|y_{ai}-y_{i}|}{\max\left(y_{i}\right)-\min\left(y_{i}\right)}\times 100\%$$

(11) $$NRMSE=\frac{\sqrt{\frac{1}{M}\sum {\left(y_{ai}-y_{i}\right)}^{2}}}{\max\left(y_{i}\right)-\min\left(y_{i}\right)}\times 100\%$$

Given that the MHL model consists of three components, i.e., a time delay, a rate-dependent model, and a linear dynamic term, the parameter identification process of the model is divided into three distinct steps in sequence.

Step 1: A step signal is input into the FSM, and the output of the system is compared with the input signal to measure the experimental system delay, which is 68 μs, as shown in Figure 5.

**Figure 9:**
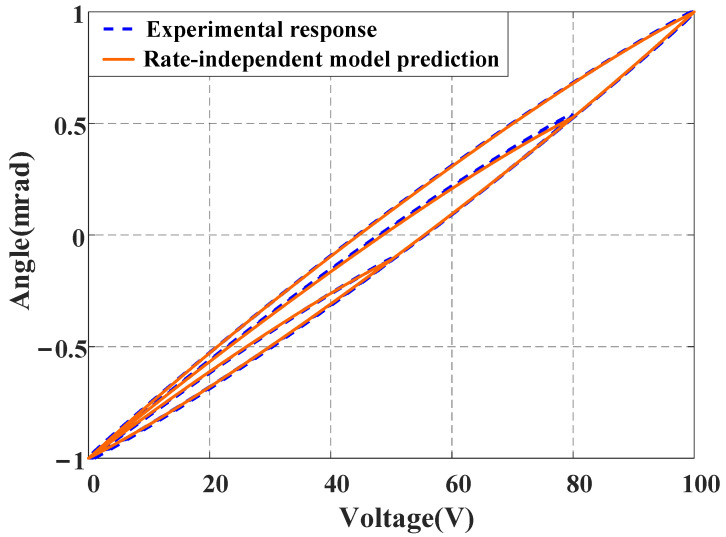
Prediction results of rate-independent hysteresis model.

Step 2: When a low-frequency signal is used as input for the FSM, its dynamic behavior is not obvious. Therefore, a variable-amplitude signal $v\left(t\right)=\frac{U}{2}[\sin(2\pi t-\frac{\pi }{2})+1]$ with $U\in \left[0,100\right]V$ is utilized to excite the FSM, and its output is obtained through the sensor. By using hysteresis curve description methods [19,20] and employing the least square method, the parameters of major hysteresis curves, as shown in Table 1, are determined. Subsequently, a rate-independent model $H_{S}\left(v\left(t\right)\right)$ and its inverse model are established. Figure 9 shows the prediction results of the established rate-independent model. It can be observed that the identified model predicts the actual angle output of the FSM very well, with an NME of 0.96% and an NRMSE of 0.25%.

To capture the dynamic behavior of the FSM within a certain frequency range, a full-amplitude frequency sweep signal $v\left(t\right)=50[\sin(2\pi ft-\frac{\pi }{2})+1]$ with frequencies $f\in \left[0,100\right]$ Hz is employed to stimulate the FSM. By performing a differential operation between the output $H_{S}\left(v\left(t\right)\right)$ of the rate-independent model and the system output $y\left(t\right)$, the output $y\left(t\right)-H_{S}\left(v\left(t\right)\right)$ of the rate correction term is acquired. By using the least square method, the parameters of the rate-dependent model and its inverse model are identified based on $y\left(t\right)-H_{S}\left(v\left(t\right)\right)$ and the input signal, as shown in Table 2. The established model is based on the phenomenological approach, where its modeling parameters bear no direct relation to the physical nature of hysteresis. Consequently, the characterization of the rate-dependent hysteresis behavior can be achieved through experimental observation data [7].

Step 3: Signals with a frequency range of $f\in \left[0,4000\right]$ Hz are applied to stimulate the FSM, and the system output $y\left(t\right)$ and the rate-dependent model output $w\left(t\right)$ are obtained. By utilizing the amplitude and phase relationship between signals $y\left(t\right)$ and $w\left(t\right)$, the parameters of the linear dynamic term $G\left(s\right)$ are further identified, as shown in Equation (12). The order and coefficients of the identified linear dynamic term $G\left(s\right)$ are determined based on the input–output data of the system and the fitting accuracy of the identification method, without necessarily providing physical insights into the modeling problems [5].

(12) $$G\left(s\right)=\frac{1.5\times 10^{4}s^{2}+1.0\times 10^{9}s+1.3\times 10^{12}}{s^{3}+2.9\times 10^{4}s^{2}+4.5\times 10^{8}s+1.3\times 10^{12}}$$

**Figure 10:**
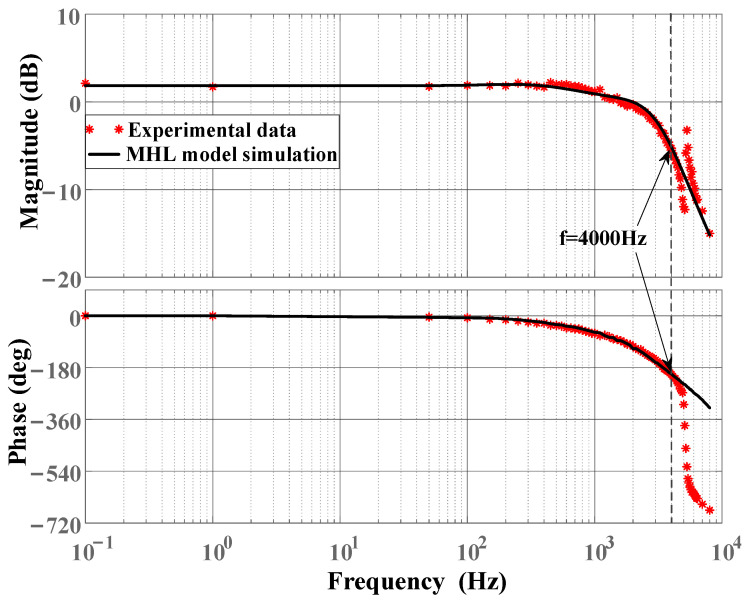
Open-loop frequency–response curve of FSM system.

Figure 10 displays the open-loop frequency–response results of the FSM system. The results indicate that the MHL model exhibits good agreement with the actual system dynamic response within the frequency range of 4000 Hz, thereby verifying the effectiveness of the identified model. Additionally, resonance phenomena are observed above 4000 Hz, accompanied by rapid phase attenuation, which is primarily attributed to unmodeled high-frequency dynamics.

It is worth mentioning that during the modeling process in step 3, the control experimental platform shown in Figure 8 is not involved. Instead, continuous and undistorted input signals are first generated by using a signal generator and then applied to the FSM. Subsequently, the undistorted output signals of the FSM are directly collected by using a high-resolution oscilloscope. Therefore, the precise acquisition of high-frequency output signals can be achieved.

### 4.3. Model Verification Results

**Figure 11:**
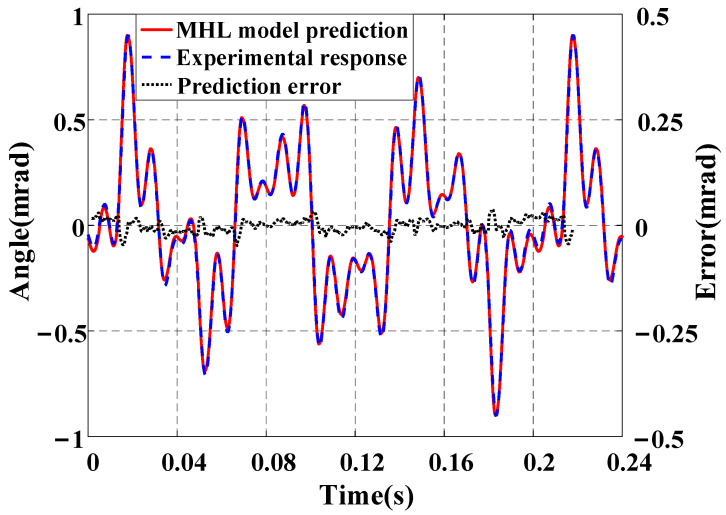
Prediction results of the MHL model under the multi-frequency input signal.

To validate the effectiveness of the MHL model, a multi-frequency signal $v\left(t\right)=50$$-19.2\sin\left(30\pi t\right)-10.7\sin\left(80\pi t\right)-10.7\sin\left(150\pi t\right)-10.7\sin\left(200\pi t\right)$, which contains frequency components of 15 Hz, 40 Hz, 75 Hz, and 100 Hz, is excited into the FSM. The modeling results presented in Figure 11 indicate that the output of the rate-dependent model is consistent with the actual data, with an NME of 2.66% and an NRMSE of 0.85%. In this frequency range, the rate-dependent model can effectively describe the system dynamics, while the output of the linear dynamic term is close to 1, which is almost ineffective.

**Figure 12:**
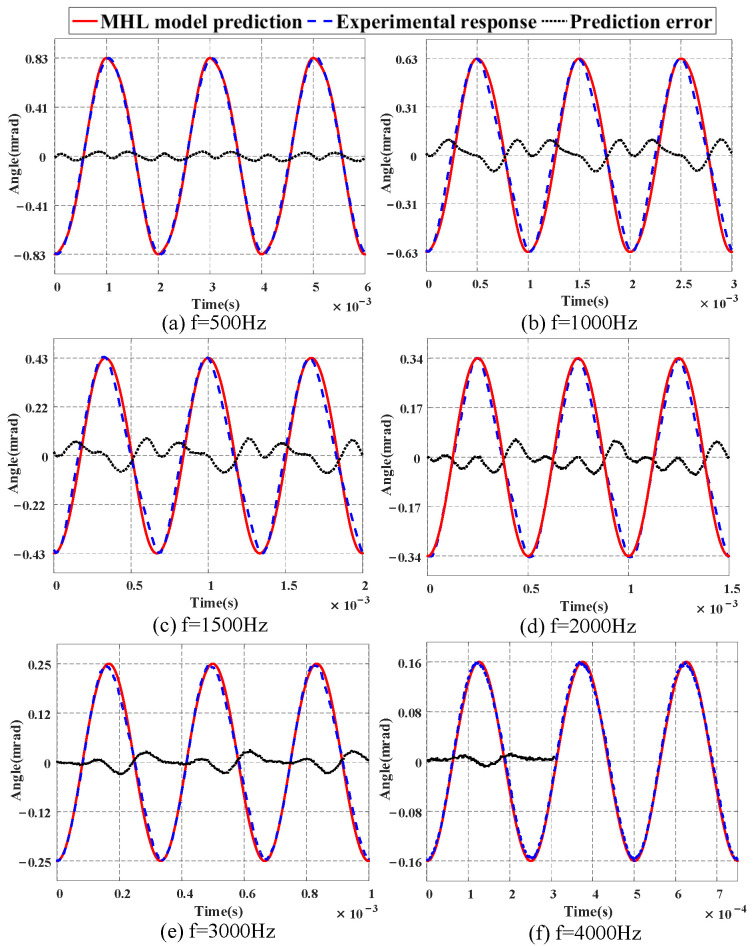
Prediction results of MHL model at different frequencies.

**Figure 13:**
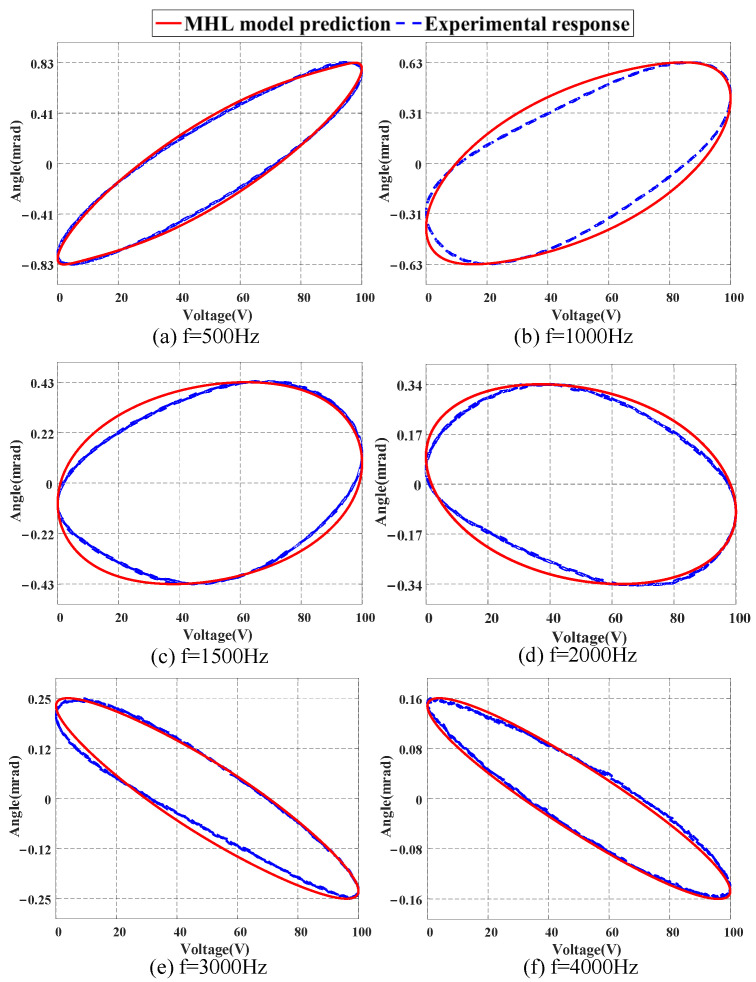
Hysteresis curves at different frequencies.

Figure 12 displays the prediction results of the MHL model within the range of 500 to 4000 Hz. It can be seen that the output of the MHL model is in good agreement with the actual response data, and due to the influence of system dynamics, the amplitude of the output signal gradually decreases as the frequency increases. Figure 13 shows the hysteresis curves at different frequencies. It can be observed that the FSM exhibits dynamic hysteresis behavior with increasing frequency, and the shape of the hysteresis curves gradually changes. From Figure 12 and Figure 13, it can be concluded that the MHL model can better describe the dynamic characteristics of the FSM within the range of 4000 Hz, demonstrating the effectiveness and feasibility of the proposed modeling method. Table 3 provides the quantitative results by listing the NME and NRMSE values.

### 4.4. Tracking Performance

**Figure 14:**
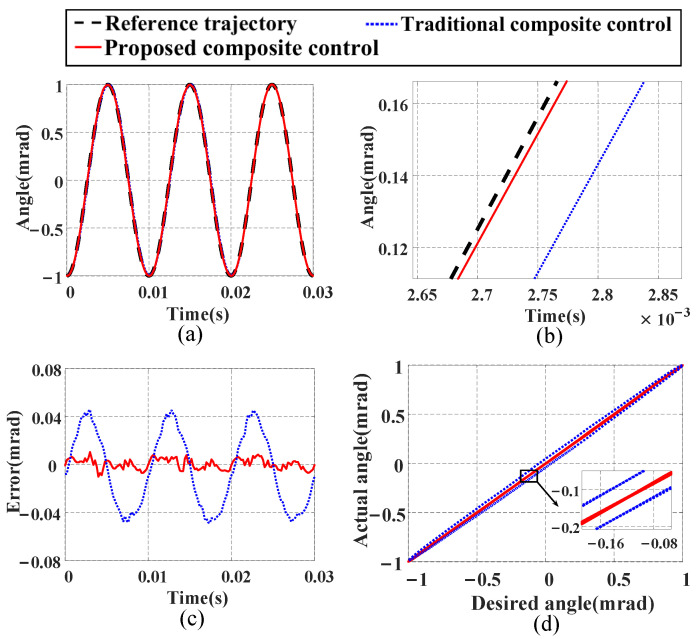
Compensation results of proposed composite control method and traditional composite control method for single-frequency input signal: (**a**) tracking curves; (**b**) enlarged view of tracking curves; (**c**) tracking errors; (**d**) hysteresis curves.

**Figure 15:**
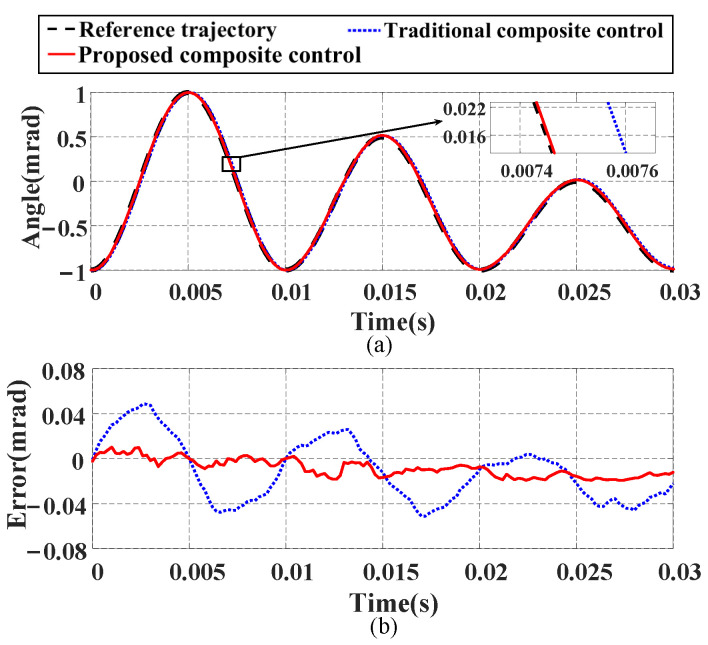
Compensation results of proposed composite control method and traditional composite control method for single-frequency decreasing input signal: (**a**) tracking curves; (**b**) tracking errors.

**Figure 16:**
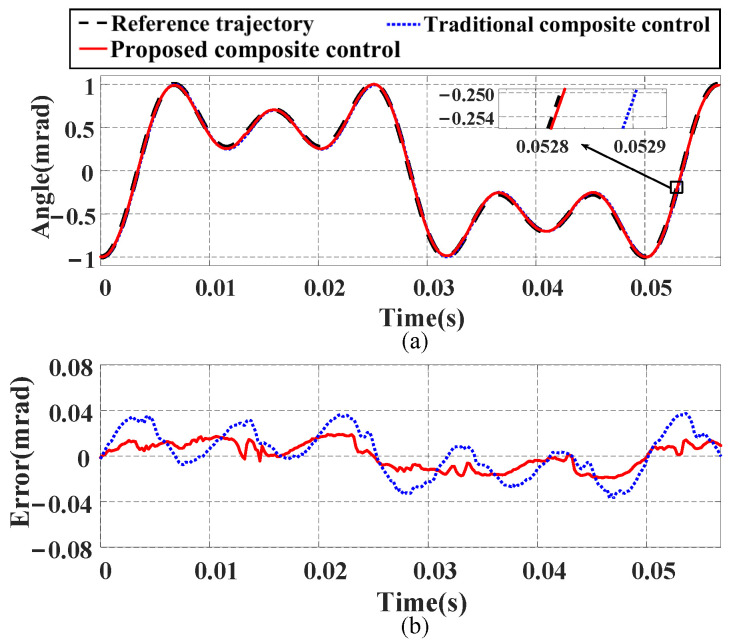
Compensation results of proposed composite control method and traditional composite control method for multi-frequency input signal: (**a**) tracking curves; (**b**) tracking errors.

In this section, three different input signals are used to compare the tracking performance of the proposed composite control method and the traditional composite control method:

(1) Tracking performance under a single-frequency signal: We input a 100 Hz sinusoidal signal as the reference trajectory for the FSM, and Figure 14 shows the tracking results. From Figure 14a–c, it can be seen that the proposed composite control method exhibits higher tracking accuracy and smaller tracking errors. Compared with the traditional composite control method, the NME is reduced by 78.6%, and the NRMSE is decreased by 86.16%. Additionally, Figure 14d shows that the proposed composite control method maintains high linearity, indicating its superiority over the traditional composite control method in tracking a single-frequency signal and proving its good tracking performance.
(2) Tracking performance under a decreasing signal: A signal with a frequency of 100 Hz and decreasing amplitude is generated to verify the performance of the proposed composite control method, with the tracking results shown in Figure 15. The experimental results demonstrate that the proposed composite control method achieves better tracking performance compared with the traditional composite control method. Specifically, the NME and NRMSE values are reduced by 62.11% and 60.54%, respectively.
(3) Tracking performance under a multi-frequency signal: We construct a multi-frequency signal $v\left(t\right)=50-35.3\sin\left(40\pi t\right)-19.6\sin\left(120\pi t\right)-19.6\sin\left(200\pi t\right)$ containing frequency components of 20 Hz, 60 Hz, and 100 Hz to verify the tracking ability of both methods for complex signals. The results shown in Figure 16 indicate that the proposed composite control method has better tracking ability. Numerically, compared with the traditional composite control method, the NME and NRMSE values are reduced by 48.66% and 43.14%, respectively. The tracking errors of both methods under the three test signals are detailed in Table 4.

**Figure 17:**
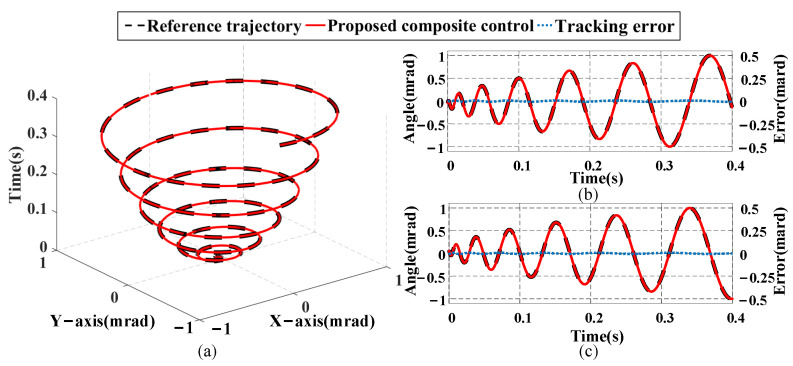
Tracking results of dual-axis spiral trajectory for FSM: (**a**) dual-axis spiral scan curves; (**b**) *x*-axis tracking results; (**c**) *y*-axis tracking results.

From the aforementioned experimental results, it is evident that the proposed composite control method exhibits superior tracking performance compared with the traditional composite control method. This further validates the effectiveness and feasibility of introducing a rate correction term into the feedforward compensator. In addition, to test the tracking ability of the proposed method for dual-axis spiral scanning signals, a spiral scanning motion [2] simultaneously conducted along the *x*- and *y*-axes within a range of −1 to 1 mrad is employed. The tracking results shown in Figure 17 demonstrate that the proposed composite control method tracks the expected motion of dual-axis spiral scanning very well.

**Figure 18:**
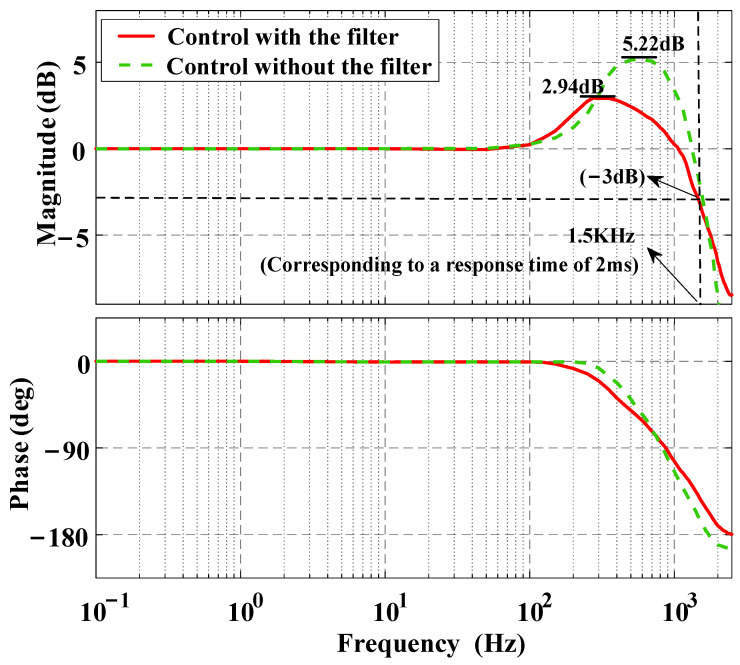
Frequency–response curves of closed-loop control system.

Although the proposed composite control method is designed as shown in Figure 6, in practical applications, a second-order filter needs to be connected in series after the rate correction term. The purpose of doing so is to suppress the peak of the amplitude–frequency characteristic curve across the entire closed-loop bandwidth range and minimize the impact on the system phase within the range of interest. The expression for this filter is $N\left(s\right)=1/\left(3.23\times 10^{-7}s^{2}+9.1\times 10^{-4}s+1\right)$, and the discretization operation is employed during the specific experimental process. When a 100 Hz signal is input to excite the FSM, the open-loop system exhibits a phase lag of $14.4^{\circ }$, the rate correction term introduces a phase lead of $61^{\circ }$, and the filter itself contributes a phase lag of $39^{\circ }$, resulting in a phase lag of $0.69^{\circ }$ for the closed-loop control system. The closed-loop frequency–response curve for the proposed composite control method is shown in Figure 18. It can be observed that the introduction of the filter has little effect on the amplitude and phase of the 100 Hz signal. Before cascading the filter, the peak value of the amplitude–frequency characteristic curve is 5.22 dB. After introducing the filter, this peak value is decreased to 2.94 dB.

### 4.5. Discussion

In the MHL model, both the rate-dependent term and the dynamic term possess the ability to describe dynamic characteristics. Therefore, defining the frequency range for each term becomes an important question. For the rate-dependent term, a corresponding inverse model can be established and utilized for feedforward compensation to achieve favorable open-loop frequency characteristics. Based on this line of thinking, it would be ideal to have the rate-dependent term describe as wide a frequency range as possible. However, in reality, as the frequency range described by the rate-dependent term widens, its modeling accuracy decreases. Thus, a more reasonable method is to design the rate-dependent term based on the system’s application requirements. Taking FSM modeling as an example, its application background is satellite laser communication. Since the vibration spectrum of satellite platforms is mainly concentrated below 100 Hz, if the rate-dependent term can cover the frequency range up to 100 Hz, the response characteristics within this frequency band can be significantly improved during inverse compensation. As shown in Figure 18, at 100 Hz, the closed-loop system exhibits an amplitude–frequency characteristic of 0.2 dB and a phase angle delay of $0.69^{\circ }$, indicating that the closed-loop system can effectively track signals below 100 Hz and suppress disturbances within this frequency range.

This study investigates a fast steering mirror (FSM) system employing a two-dimensional (*x*-/*y*-axes) piezoelectric actuation architecture for satellite laser communication beam control, achieving a 2 mrad deflection range with a 0.05 μrad resolution. As shown in Figure 18, the proposed composite control method demonstrates a closed-loop bandwidth of 1.5 KHz and a settling time of 2 ms. The tracking test results in Section 4.4 reveal outstanding control performance with a normalized maximum error (NME) consistently below 1% and a normalized root-mean-square error (NRMSE) maintained within 0.6% under various test signals, confirming the superior capabilities in high-bandwidth, rapid-response, and high-precision control. For satellite laser communication applications, the positioning accuracy of the beam control system may be affected by two primary factors: temperature-induced parameter drift in the piezoelectric ceramics and external mechanical disturbances from satellite platform vibrations. However, the temperature dependence of piezoelectric parameters typically exhibits slow time-varying characteristics, while platform vibration energy is predominantly concentrated below 100 Hz. Consequently, the developed composite control method with a 1.5 KHz closed-loop bandwidth effectively suppresses both the temperature-dependent parameter variations and platform vibration disturbances below 100 Hz.

The MHL model identified in the experiments is constructed based on an FSM system equipped with a high-voltage driver. Due to the integration of a notch filter in the driver, the frequency characteristics of the signal within the relevant range are adjusted. This results in the resonance phenomenon, originally anticipated near 2 kHz, not being displayed in Figure 10. This also explains why the modeling errors in Figure 12 and Figure 13 do not exhibit a monotonically increasing trend with the input frequency. However, it should be clarified that this adjustment does not alter the inherent resonant frequency of the FSM system itself.

It is noteworthy that compared with the rate-dependent hysteresis model presented in [23], the proposed model, which combines the time delay term and the linear dynamic term, effectively describes the dynamic characteristics of the system in the frequency range of 4 kHz. This model not only extends the modeling method in [23] but also addresses the inherent limitations of its rate-dependent model. Furthermore, we tailor a composite control method for the proposed model, which falls into the category of closed-loop control. The compensation method mentioned in [23] belongs to open-loop control and is only a part of our composite controller.

In the composite controller designed for MHL, this time delay is not specifically addressed because a 68 μs delay accounts for only one-third of the 200 μs sampling time. In subsequent research, we will adopt the Dahlin algorithm or predictive control algorithm to compensate for the time delay term in order to achieve better control performance.

## 5. Conclusions

This paper proposes a modified Hammerstein-like (MHL) model which consists of an input time delay, a rate-dependent hysteresis term, and a linear dynamic term in a cascaded structure, effectively describing the dynamic characteristics of FSM systems over a wide frequency range. Furthermore, a composite control approach tailored for the established model is introduced. The presented controller consists of a feedforward compensator and a proportional–integral (PI) controller. The former is the inverse of the rate-dependent term in the MHL model, while the latter is used for closed-loop regulation. Experimental results validate the effectiveness and feasibility of the proposed modeling and composite control methods. This study provides novel and effective methods for modeling and compensating piezoelectric systems, with potential applications in a wider range of fields. In future work, we will explore the application of advanced control algorithms to construct feedback controllers with the goal of achieving greater efficient composite control performance.

## Figures and Tables

**Table 1:** Model and inverse model parameters of $f_{0}\left(x\right)$ and $g_{0}\left(x\right)$.

	Model	Inverse Model
$\left(p_{5},q_{5}\right)$	(−0.4624,0.9457)	(0.4523,−0.04333)
$\left(p_{4},q_{4}\right)$	(1.788,−3.005)	(−1.422,0.3898)
$\left(p_{3},q_{3}\right)$	(−2.398,3.248)	(1.7,−0.5418)
$\left(p_{2},q_{2}\right)$	(2.037,−2.083)	(−1.125,0.4221)
$\left(p_{1},q_{1}\right)$	(2.228,4.104)	(1.397,0.7696)

**Table 2:** Parameters of $H_{D}\left(v\left(t\right)\right)$ and its inverse.

	Model	Inverse Model
$k_{1}$	−0.001265	0.00036
$b_{1}$	−0.021	−0.0005
$k_{2}$	0.0007	0.000522
$b_{2}$	1	1

**Table 3:** MHL model verification results at different frequencies.

Frequency	500 Hz	1000 Hz	1500 Hz	2000 Hz	3000 Hz	4000 Hz
NME	2.65%	8.47%	8.96%	8.99%	6.28%	4.29%
NRMSE	1.46%	4.84%	4.88%	4.44%	2.85%	1.87%

**Table 4:** Tracking results of different control methods.

Types of	Traditional Composite Control	Proposed Composite Control
Reference Trajectories	(NME/NRMSE)	(NME/NRMSE)
Single-frequency signal	2.43%/1.59%	0.52%/0.22%
Decreasing signal	2.56%/1.47%	0.97%/0.58%
Multi-frequency signal	1.87%/1.02%	0.96%/0.58%

## Data Availability

The data that support the findings of this study are available from the corresponding author upon reasonable request.

## References

[1] Wang G., Wang Y., Zhou H., Bai F., Chen G., Ma J. (2019). Comprehensive approach to modeling and identification of a two-axis piezoelectric fast steering mirror system based on multi-component analysis and synthesis. Mech. Syst. Signal Proc..

[2] Cao K., Du H., Zhang J., Hao G., Ran Q., Ma J. (2023). Calculation of average acquisition probability for spiral–circular composite scanning in free space optical communication. Opt. Commun..

[3] Jiles D.C., Atherton D.L. (1986). Theory of ferromagnetic hysteresis. J. Magn. Magn. Mater..

[4] Rosenbaum S., Ruderman M., Strohla T., Bertram T. (2010). Use of JilesAtherton and Preisach hysteresis models for inverse feed-forward control. IEEE Trans. Magn..

[5] Gu G., Zhu L., Su C., Ding H., Fatikow S. (2016). Modeling and Control of Piezo-Actuated Nanopositioning Stages: A Survey. IEEE Trans. Autom. Sci. Eng..

[6] Zou J., Gu G. (2019). High-Precision Tracking Control of a Soft Dielectric Elastomer Actuator With Inverse Viscoelastic Hysteresis Compensation. IEEE-ASME Trans. Mechatron..

[7] Ang W., Khosla P., Riviere C. (2007). Feedforward Controller With Inverse Rate-Dependent Model for Piezoelectric Actuators in Trajectory-Tracking Applications. IEEE-ASME Trans. Mechatron..

[8] Li Z., Fan X., Long D., Yang Z., Shi C. (2025). A Modular Direct Description Method With Low Memory Usage and Execution Time for Hysteresis Modeling and Compensation of Piezoelectric Actuators. IEEE Trans. Autom. Sci. Eng..

[9] Zhu W., Wang D. (2012). Non-symmetrical Bouc–Wen model for piezoelectric ceramic actuators. Sens. Actuator A-Phys..

[10] Wang G., Chen G., Bai F. (2015). Modeling and identification of asymmetric Bouc–Wen hysteresis for piezoelectric actuator via a novel differential evolution algorithm. Sens. Actuator A-Phys..

[11] Ahmed K., Yan P., Li S. (2021). Duhem Model-Based Hysteresis Identification in Piezo-Actuated Nano-Stage Using Modified Particle Swarm Optimization. Micromachines.

[12] Gan J., Mei Z., Chen X., Zhou Y., Ge M. (2019). A Modified Duhem Model for Rate-Dependent Hysteresis Behaviors. Micromachines.

[13] Dong Y., Hu H., Wang H. (2014). Identification and experimental assessment of two-input Preisach model for coupling hysteresis in piezoelectric stack actuators. Sens. Actuator A-Phys..

[14] Gu G., Zhu L., Su C. (2014). Modeling and Compensation of Asymmetric Hysteresis Nonlinearity for Piezoceramic Actuators With a Modified Prandtl–Ishlinskii Model. IEEE Trans. Ind. Electron..

[15] Zhou C., Feng C., Aye Y., Ang W. (2021). A Digitized Representation of the Modified Prandtl–Ishlinskii Hysteresis Model for Modeling and Compensating Piezoelectric Actuator Hysteresis. Micromachines.

[16] Liu Y., Du D., Qi N., Zhao J. (2019). A Distributed Parameter Maxwell-Slip Model for the Hysteresis in Piezoelectric Actuators. IEEE Trans. Ind. Electron..

[17] Delibas B., Arockiarajan A., Seemann W. (2006). Rate dependent properties of perovskite type tetragonal piezoelectric materials using micromechanical model. Int. J. Solids Struct..

[18] Vaiana N., Sessa S., Marmo F., Rosati L. (2018). A class of uniaxial phenomenological models for simulating hysteretic phenomena in rate-independent mechanical systems and materials. Nonlinear Dyn..

[19] Cao K., Li R. (2019). Modeling of Rate-Independent and Symmetric Hysteresis Based on Madelung’s Rules. Sensors.

[20] Li R., Cao K., Yu X., Zeng M. (2021). Modeling and Compensation Algorithms of Asymmetric Nonlinearity for Piezoelectric Actuators Based on Madelung’s Rules. IEEE Trans. Ind. Electron..

[21] Al Janaideh M., Rakheja S., Su C. (2009). Experimental characterization and modeling of rate-dependent hysteresis of a piezoceramic actuator. Mechatronics.

[22] Al Janaideh M., Krejčí P. (2013). Inverse Rate-Dependent Prandtl–Ishlinskii Model for Feedforward Compensation of Hysteresis in a Piezomicropositioning Actuator. IEEE-ASME Trans. Mechatron..

[23] Hao G., Cao K., Li R., Li Z., Du H., Tan L. (2024). Rate-dependent hysteresis modeling and compensation for fast steering mirrors. Sens. Actuator A-Phys..

[24] Gao Z., Wang Y., Shao M., Zhu X. (2022). Theoretical and experimental investigation study of discrete time rate-dependent hysteresis modeling and adaptive vibration control for smart flexible beam with MFC actuators. Sens. Actuator A-Phys..

[25] Li W., Liu K., Yang Z., Wang W. (2022). Dynamic modeling and disturbance rejection compensation for hysteresis nonlinearity of high voltage piezoelectric stack actuators. Smart Mater. Struct..

[26] Wadikhaye S., Yong Y., Bhikkaji B., Moheimani S. (2014). Control of a piezoelectrically actuated high-speed serial-kinematic AFM nanopositioner. Smart Mater. Struct..

[27] Gu G., Li C., Zhu L., Su C. (2016). Modeling and Identification of Piezoelectric-Actuated Stages Cascading Hysteresis Nonlinearity With Linear Dynamics. IEEE-ASME Trans. Mechatron..

[28] Zhang Z., Yan P. (2021). Enhanced robust nanopositioning control for an X-Y piezoelectric stage with sensor delays: An infinite dimensional *H*_∞_ optimization approach. Mechatronics.

[29] Chai G., Tan Y., Tan Q., Dong R., Long X. (2024). Predictive Gradient Based Control Using Hammerstein Model for MEMS Micromirrors. IEEE-ASME Trans. Mechatron..

[30] Zirka S., Moroz Y. (1995). Hysteresis modeling based on transplantation. IEEE Trans. Magn..

[31] Zirka S., Moroz Y. (1999). Hysteresis modeling based on similarity. IEEE Trans. Magn..

